# Multi-trait diversification in marine diatoms in constant and warmed environments

**DOI:** 10.1098/rspb.2023.2564

**Published:** 2024-03-27

**Authors:** Jana Hinners, Phoebe A. Argyle, Nathan G. Walworth, Martina A. Doblin, Naomi M. Levine, Sinéad Collins

**Affiliations:** ^1^ Helmholtz-Zentrum Hereon, Max-Planck-Straße 1, 21502 Geesthacht, Germany; ^2^ Institute of Ecology and Evolution, University of Edinburgh, Charlotte Auerbach Road, Edinburgh, EH9 3FL, UK; ^3^ Climate Change Cluster, University of Technology Sydney, Sydney, New South Wales 2007, Australia; ^4^ Department of Biological Sciences, University of Southern California, Los Angeles, CA 91011, USA; ^5^ Sydney Institute of Marine Science, Mosman, New South Wales 2088, Australia

**Keywords:** phytoplankton, experimental evolution, diatom, functional traits, warming

## Abstract

Phytoplankton are photosynthetic marine microbes that affect food webs, nutrient cycles and climate regulation. Their roles are determined by correlated phytoplankton functional traits including cell size, chlorophyll content and cellular composition. Here, we explore patterns of evolution in interrelated trait values and correlations. Because both chance events and natural selection contribute to phytoplankton trait evolution, we used population bottlenecks to diversify six genotypes of Thalassiosirid diatoms. We then evolved them as large populations in two environments. Interspecific variation and within-species evolution were visualized for nine traits and their correlations using reduced axes (a trait-scape). Our main findings are that shifts in trait values resulted in movement of evolving populations within the trait-scape in both environments, but were more frequent when large populations evolved in a novel environment. Which trait relationships evolved was population-specific, but greater departures from ancestral trait correlations were associated with lower population growth rates. There was no single master trait that could be used to understand multi-trait evolution. Instead, repeatable multi-trait evolution occurred along a major axis of variation defined by several diatom traits and trait relationships. Because trait-scapes capture changes in trait relationships and values together, they offer an insightful way to study multi-trait variation.

## 1. Introduction

Phytoplankton functional traits such as cell size, elemental composition and population growth rates are crucial to the roles that phytoplankton play in ocean ecology and biogeochemical cycling. Functional traits are highly diverse across photosynthetic marine microbes (phytoplankton), allowing them to thrive in all aquatic environments. This diversity evolves both by natural selection and chance events [1]. This tension between the actions of natural selection and chance is often framed as the roles of local adaptation versus migration in determining phytoplankton distributions and trait diversity [2–4]. However, our understanding of how interrelated functional traits can diversify has focused almost exclusively on adaptation to changing environments and rarely has incorporated variation fixed through chance events. Modelling, predicting and explaining functional trait evolution requires defining the patterns of viable variation in functional traits generated during chance events, such as founder effects that can occur during migration or at the start of diatom bloom events, since this variation can be what natural selection acts on. Understanding how interrelated functional traits evolve, both in the absence of environmental change and because of it, is vital for understanding patterns of trait change in phytoplankton and subsequent effects on ecology and biogeochemistry.

Phytoplankton diversity is often framed in terms of variation in phytoplankton functional traits, such as cell size [5,6]. Functional traits of diatoms are well studied, and relationships between functional traits are usually considered as pairwise correlations, often involving fundamental trade-offs between traits, such as cell size and nutrient uptake affinity [7,8]. Based on this, trait-based models of diatoms and other phytoplankton often use the simplifying assumption that fitness is determined by a single master trait (e.g. size) and that all other traits are linked through a fixed correlation with the master trait [5]. This therefore assumes that trait correlations are maintained during evolution, and that pairwise correlations are sufficient to describe constraints on complex phenotypes. However, multi-trait relationships that extend beyond pairwise correlations can constrain phenotypic change on both plastic [9,10] and evolutionary [11] time scales. In addition, adaptive outcomes are consistent with occasional departures from ancestral trait correlations during adaptation [11]. Multi-trait relationships can be identified through statistical techniques such as principal component analyses (PCA), which can define the ‘trait-scape’ for phytoplankton phenotypes [9] in one or more environments, for a set of traits that are commonly used to understand ecological or biogeochemical function. The trait-scape collapses multi-trait variation onto a reduced set of axes, which allows us to study variation in multi-trait phenotypes rather than in single traits. By contrast to master trait approaches, trait-scapes reveal shifts in trait correlations as well as in trait values, and automatically incorporate trait correlations beyond pairwise comparisons to include statistical correlations between three or more traits, whether or not knowledge of the biological basis for trait correlations is complete or correct. It is also worth noting that single locations in the trait-scape can define more than one multi-trait phenotype [11], and that the single-trait measurements are still useful for interpreting patterns of variation in, and movement on, trait-scapes. Trait-scapes reveal constraints on multi-trait change, but trait evolution can occur without movement in trait space, so conventional measures of evolution must be used alongside trait-scapes.

Here, we used population bottlenecks to drive rapid diversification [12], which enabled us to explore patterns of evolutionary change in interrelated functional traits that affect the ecosystem and biogeochemical function of diatoms. We did this both in a constant temperature environment and under moderate warming previously shown to drive adaptation in this genus [13]. Our motivation for using population bottlenecks was twofold. First, these conditions allowed evolutionary trait diversification, a drop in fitness, and fitness recovery (adaptation) in the absence of environmental change [12], which reveals how interrelated traits can vary through periodic chance events alone. Second, although chance events have the potential to contribute to diversification in populations of marine microbes, they are rarely used to generate genetic (and associated trait) diversity for adaptation in experiments. Chance events, such as drastic reductions in population size, have the potential to affect marine microbes *in situ*. Diatoms, for example, can experience extreme fluctuations in population size both during bloom and bust growth, and as a result of the physical forces in ocean currents which can transport them across different ecoregions [14,15]. While there is ample evidence that natural selection does underlie some of phytoplankton trait diversity [16–18], it is also likely that chance events play an important role. Specifically, populations adapting to temperature changes are often doing so either as they begin to bloom, or after a migration event, both of which involve chance acting immediately prior to selection, partially determining variation that natural selection acts on.

**Figure 1.**
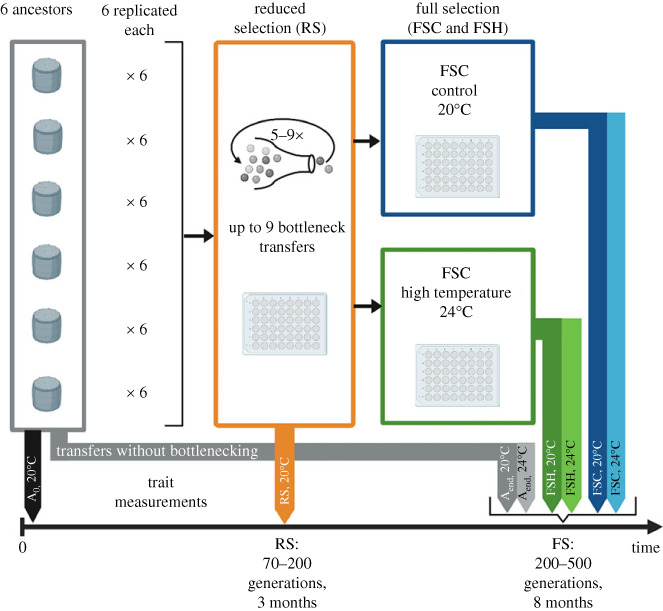
Schematic of the experimental design. Six strains (1010, 1050, 1059, 1587, 2929, and 3367) were used for the experiment. Six replicates from each strain (1010-1, -2, ...-6, 1050-1,…) underwent up to nine bottlenecks, of approximately eight cells. After that, lineages were transferred regularly for eight months under full selection with large inoculation sizes. The full selection was performed under control (1010-1C, 1010-2C,…) and high-temperature (+4°C) conditions (1010-1H, 1010-2H,…). Phenotypic trait measurements under control assay conditions took place at the beginning of the experiment (from the ancestral strains, A_0_), and at the end of the reduced-selection phase (RS). At the end of the full-selection phase, trait measurements of all ancestral (A_end_), evolved control (FSC) and high-temperature lineages (FSH) were performed under control and high-temperature assay conditions.

We used repeated population bottlenecks to increase genetic and phenotypic divergence between populations of diatoms. The experiment used six genetically and phenotypically distinct strains, in replicate (figure 1 for schematic). For clarity, we refer to each starting genotype as a strain and to each evolving replicate population that is related by descent as a lineage. During bottlenecks, relaxed stabilizing selection allows new multi-trait phenotypes to emerge. This is the reduced-selection (RS) phase of the experiment. Most mutations accumulated during mutation accumulation experiments are deleterious. Thus, many RS populations have lower population growth rates than their ancestors because chance, rather than natural selection, plays a dominant role in which cells survive. Using population bottlenecks to generate diversity in the form of divergence between replicate populations is the basis of mutation accumulation experiments [19], which are commonly used in experimental evolution. Following the RS phase, we observed how natural selection acts on this trait variation during fitness recovery by evolving the lineages as large populations [20], where natural selection is more effective, allowing more fit multi-trait phenotypes to increase in frequency within each population during the full-selection (FS) phase of the experiment. Because population bottlenecks erode variation within populations, evolution in the FS phase of the experiment can be driven by de novo heritable changes, as is usual in microbial evolution experiments of this scale. The experiment was ended when population growth rates stabilized, indicating that (i) that FS populations were on or near local phenotypic optima, and that further adaptation would probably require an input of further novel variation that does not arise rapidly [21], or (ii) that natural selection was acting on small differences in fitness, and increases in fitness would be slow relative to time scales in nature such as diatom blooms or growing seasons.

This study assessed movement on trait-scapes for populations subjected to both chance and natural selection with and without environmentally driven adaptation in the form of a temperature increase (see electronic supplementary material, box 1). The findings provide insight into patterns of multi-trait variation in diatoms at multiple taxonomic levels, environments and time scales. We also address the role of departures from ancestral trait correlations during multi-trait evolution, both in the absence and presence of environmental change, and contextualize the role of evolving departures from ancestral trait correlations in terms of effects on growth rates and movement in trait space. We demonstrate that novel phenotypes emerged as a result of natural selection acting on variation generated by chance events even in the absence of environmental change.

## 2. Results

### (a) Effectiveness of population bottlenecks and fitness recovery

The goal of our study was to examine patterns of variation generated in multi-trait phenotypes in diatoms. We assume that our ancestral populations were well adapted to control conditions. Our rationale for this assumption is that the ancestral strains were all from culture collections and had been grown in similar laboratory conditions in our laboratory for at least five months before the experiment, during which time they had stable population growth rates. The population bottlenecks resulted in growth rate fluctuations and an average reduction in population growth rate of 17–65% by the end of the RS phase compared with ancestral rates (electronic supplementary material, figure S3). Large reductions in population growth rate in batch culture probably negatively impact fitness, suggesting that the RS populations were low fitness. In addition, the RS populations had different phenotypes from their ancestors in the ancestral environment (electronic supplementary material, figure S6), indicating that the changes seen are not attributable to a plastic response, as there was no environmental change to respond to. Following population bottlenecks, full-selection populations under control (FSC) and high-temperature conditions (FSH) evolved higher population growth rates again that stabilized on average 20% below the growth of ancestral populations (electronic supplementary material, figure S4; *F*_1,143_ = 111, *p* < 0.01) maintained under control conditions as large populations. This increase in growth rate between the RS populations and the FS populations indicates effective selection on rapid growth during the FS phase of the experiment.

### (b) The trait-scape

Previous work demonstrated that the ancestral multi-trait *Thalassiosira* phenotypes could be studied using reduced axes (a trait-scape) that captured variation between genotypes [9,22]. We leveraged this understanding to quantify key traits that were orthogonal to each other and thus allowed us to identify the multi-trait trait-scape. Here, we used the trait-scape to assess patterns of variation generated within evolving single-genotype populations.

**Figure 2.**
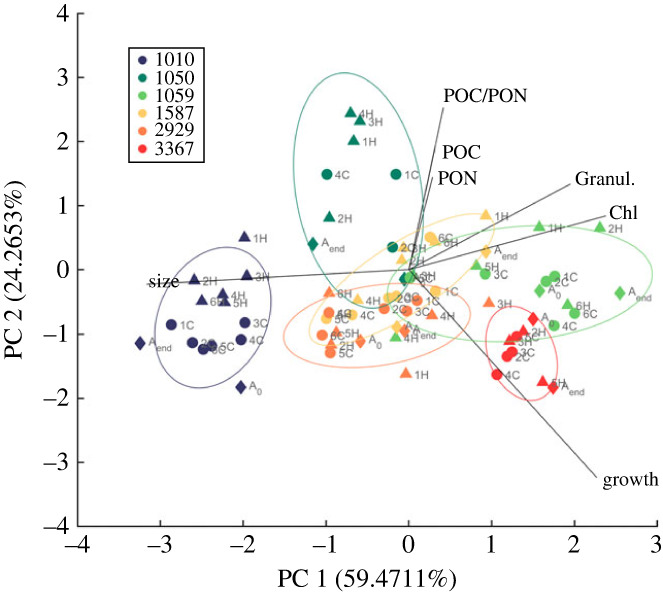
The trait-scape based on the principal component analysis of all ancestral (A_0_ and A_end_) and evolved (FSC and FSH) populations. Trait values were obtained under control conditions. Ancestors, FSC and FSH evolved populations are visualized together, colour-coded by strain. Ancestors are marked with a diamond, FSC populations with a circle, and FSH populations with a triangle. Replicates from the reduced selection are indicated by numbers; A_0_ and A_end_ mark the ancestral populations measured at the beginning and end of the experiment. Circles represent 75% confidence intervals for each strain.

To assess movement across the trait-scape, we combined the ancestral and evolved populations into one PCA (figure 2). The first two principal components of the trait-scape explain almost 85% of the variation in trait values of nine functional traits (population growth rate, cell size, cell complexity, chlorophyll a content, particulate organic carbon and nitrogen, lipid content, silicic acid uptake, intracellular reactive oxygen; see Material and methods). Interspecific variation across strains (indicated by different colours) was significantly larger than within-strain variation over the course of the experiment (*F*_1,11338_ = 1256, *p* < 0.01), confirming that the trait-scape constructed from ancestral and evolved populations captured intraspecific variation. This was in good agreement with previous experiments using these ancestral strains [9].

Most strains stayed on or returned to their ancestral location in the trait-scape after bottlenecks and subsequent evolution as large populations. This suggests that short periods of lineage evolution, such as would occur during individual blooms or growing seasons, do not usually overcome intragenus variation in these environments. Overall, the trait-scape was evenly filled by the six strains, without large gaps that would indicate non-allowable trait value combinations within the space defined by the first two principal components. This result is consistent with previous experiments with these strains [9]. Below we focus on multi-trait phenotype change. Multi-trait change is the focus of this study; changes in single traits across the experiment, changes in pairwise trait relationships, and the movement of strains in the trait-scape including all reduced-selection stages are in electronic supplementary material, figures S6 and S7.

### (c) Movement of evolving populations within the trait-scape

We used the movement of populations on the trait-scape to define patterns of change to multi-trait phenotypes in two environments. FSC populations evolved in the ancestral environment so that any phenotypic variation between replicate populations of the same genotype was the result of population bottlenecks and fitness recovery only. FSH populations evolved in a warmed environment, where environmentally imposed selection during fitness recovery could also contribute to movement on the trait-scape. We categorized movement in the trait-scape for each lineage as revealing either an isolated peak, a connected peak, or a novel peak (see electronic supplementary material, box 1 and §5(e)(iii)).

**Figure 3.**
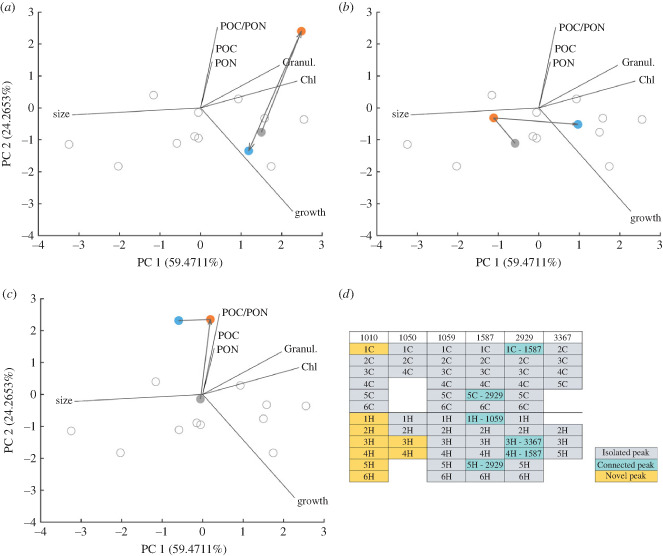
Example movements of three populations (3367-2C, 2929-3H and 1050-3H) in the trait-scape (*a–c*). The principal component analysis was performed based on trait data from ancestral, FSC and FSH populations; reduced-selection populations were projected into this trait-scape. Data from all ancestors (A_0_ and A_end_) are depicted in grey, unfilled circles. Arrows follow the movements from an ancestral population (A_0_, grey filled circle) to its location after reduced selection (orange), and then to its location after full selection (blue). (*d*) Categorization of all populations into isolated, connected or novel peaks. in the case of connected peaks, the connected strain is mentioned. Populations are sorted by strains, numbers represent reduced-selection replicate identities, and letters indicate full-selection conditions (C, control; or H, high temperature).

Despite most evolving populations returning to their ancestral location in the trait-scape, we observed all possible types of movement in the trait-scape (electronic supplementary material, box 1, figure 3), with evidence for isolated, connected and novel peaks (figure 3*d*). When considering both FSC and FSH populations (62 populations total), nine of the 62 populations (14.5%) moved to a novel peak in the trait-scape, six of the 62 (6.7%) moved to a connected peak and 47 of the 62 (75.8%) returned to their ancestral peak. Exploration of the trait-scape was limited, but not absent, for FSC populations. Of the 31 FSC populations, only one population found a novel peak and only two populations moved to a connected peak. The two populations that found connected peaks were reciprocal—a single pair of peaks were connected on this landscape during our experiment (1587–2929). This indicates that, first, the trait-scape is reasonably well sampled in the ancestral environment, and second, that it is possible for lineages to move on the trait-scape and shift multi-trait phenotypes due to demographic perturbations alone.

Changes in multi-trait phenotypes were more widespread when populations evolved in a different environment from their ancestor. Of the 31 FSH populations, four found connected peaks, evidencing three pairs of connected peaks on the trait-scape (1587–2929, 1587–1059, 3367–2929). Eight FSH populations from two starting genotypes located novel peaks, with strain 1010 often finding novel peaks. However, even when populations evolved in warmed environment, the ancestral locations in the trait-scape captured a large proportion of the locations occupied by the FSH populations—only two of the six strains located novel peaks on the trait-scape, though all six strains evolved novel multi-trait phenotypes (i.e. different values for some of the single traits; see electronic supplementary material, figure S7). This is surprising, given that warming is known to drive both plastic and evolutionary responses in *Thalassiosira*, so we expected more novel peaks [13,18]. Our data suggest that the outcome of adaptation to moderate warming, including shifts in multi-trait phenotypes, varies between strains, and between replicate populations (electronic supplementary material, figure S7). However, in this experiment, 19 out of 31 FSH populations (61%) returned to their ancestral location in the trait-scape.

### (d) Patterns of multi-trait evolution

Population bottlenecks transiently relax the efficacy of natural selection, with the potential for rapid departures from ancestral trait values and relationships. While most of the evolving populations returned to their ancestral locations in the trait-scape during fitness recovery, novel trait combinations did sometimes occur and persist in FS populations over the course of this experiment.

Strains in locations categorized as isolated peaks in the trait-scape (1059 and 3367), showed no change from ancestral trait values when single traits were compared for the ancestral and FS populations of the same genotype (electronic supplementary material, figure S7). Thus, even though many lineages evolved trait values different from their ancestors when selection was relaxed (RS phase) (electronic supplementary material, figures S6 and S7), most re-evolved ancestral trait values when selection was restored (FS phase). We were not able to measure all traits in the RS populations due to low growth and subsequent low biomass in these populations, so that changes in reactive oxygen species (ROS), silica uptake and lipid content do not contribute to patterns on trait-scapes that include RS populations.

**Figure 4.**
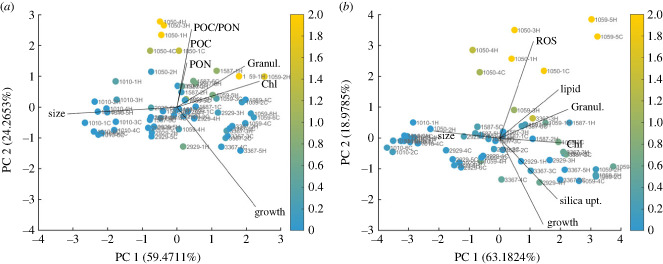
The trait-scape of all FSC and FSH population measured under control (*a*) and high temperature (*b*) conditions, with outliers from ancestral trait correlations indicated by colour. Traits included in the principal component analysis differed between the two temperature conditions to include as many traits as possible, while maintaining comparability to the trait-scape that was calculated based on the available trait data of all ancestral, reduced-selection and evolved lineages.

**Figure 5.**
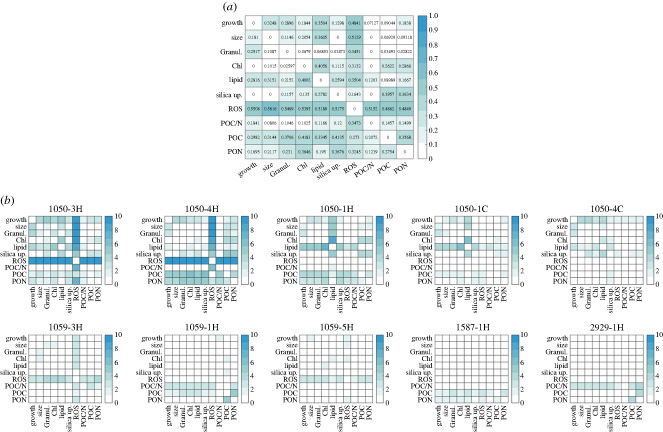
Outlier analysis for all pairwise trait correlations across strains (*a*) and for the 10 strongest outliers (*b*).

To categorize peaks using the full set of traits available in this study, we repeated the categorization above using only the ancestral and FS populations, so that the full set of traits could be used (figures 4 and 5, electronic supplementary material, figure S9). We found large increases in ROS in lineages 1050-3H and 1050-4H, suggesting that our categorization of movement based on a trait-scape that includes the RS populations, where ROS was not measurable, may underestimate novel peaks. Transcriptomic analysis of the FSC populations evolved in the ancestral environment also found increased relative expression levels of transcripts related to ROS, carbon metabolism and nitrogen metabolism [23].

Interestingly, populations moving between connected peaks in the trait-scape did not evolve the same set of trait values as the ancestral strain originally on that peak (electronic supplementary material, figures S11 and S12). Instead, they developed multi-trait phenotypes distinct from both their own ancestor and the ancestors or FS populations of other strains. This agrees with simulations of adaptation on trait-scapes [11] that predict the evolution of cryptic phenotypes, where more than one multi-trait phenotype exists in a single location in trait-scape. This is exciting, because while novel multi-trait phenotypes evolved, trait evolution was captured within the pre-existing trait-scape and often involved moving to a location in the trait-scape occupied by a different strain. The number of possible multi-trait phenotypes present in a single location in the trait-scape could be reduced by including more traits that load on the first two axes of the trait-scape. Candidates for additional traits should vary between strains or species, and depend on environmental changes under investigation. For changing temperature, respiration is one potential candidate trait [24].

### (e) The role of trait correlations in the evolution of novel phenotypes

While a change of location in the trait-scape results from a shift in trait values, trait relationships can play a key role in the evolvability of phenotypes and the resulting multi-trait phenotype [11]. For most of the pairwise trait correlations examined, evolved populations maintained the ancestral trait relationship (see electronic supplementary material, figure S8). However, a few individual populations deviated from ancestral correlations. To examine whether particular trait values or correlations were associated with departures from the ancestral correlations, we calculated an outlier score for each FS population. This score quantifies how much the relationship between two traits shifted between the ancestral correlation and the evolved trait relationship (see §5(e)(iv)). First, we examined whether larger departures from ancestral multi-trait correlations (higher outlier scores) were associated with particular trait values. We found that some trait values were indeed associated with high outlier scores. For populations evolved in the ancestral environment (FSC), populations with high particulate organic carbon (POC) and nitrogen (PON) content, as well as high granularity and chlorophyll content depart most from ancestral trait correlations, whereas populations with large cell sizes and high growth rates depart least from ancestral trait correlations (figure 4*a*). Populations that evolved in the warmed environment (FSH) showed a similar pattern, where populations with large cell sizes and high growth rates had lower outlier scores (figure 4*b*). In FSH populations, where temperature was elevated, many populations with high outlier scores also had high ROS values. This suggests that large departures from ancestral trait correlations may be stressful for the cells (electronic supplementary material, figure S9) [25–27].

We then examined whether specific traits and associated trait correlations were more likely to evolve than others. To answer this, we compared the mean residual values across strains for each pairwise trait correlation (figure 5*a*). Nine out of the 10 most extreme outliers evolved under high temperature, indicating that evolution in a new environment may favour changes in trait correlations (figure 5*b*). Across all outliers, the largest outlier scores involved correlations with ROS, POC, PON and lipid content (figure 5*a*), although more modest departures from ancestral correlations occurred in many other pairwise trait correlations. Taken together, this indicates that high outlier scores are typically associated with a substantial and directionally consistent (i.e. increase or decrease) change in a few key traits. This means that some traits are more likely to be associated with departures from ancestral trait correlations than are others. Second, it shows that some trait correlations may evolve more readily than others.

While trait correlations evolved in most populations, the identity of the trait correlations that departed most from ancestral values were population-specific (figure 5*b*). For 1050-3H, 1050-4H, 1059-3H and 1059-5H, the correlations between ROS and other traits changed the most from the ancestral correlations. For 1050-1C and 1050-1H, the correlations between lipid content and other traits diverged most from ancestral correlations, and for 1059-1H and 1587-1H the relationship between POC and PON content changed the most from the ancestral correlations. Strains 1050 and 1059 had the greatest number of outlier populations, suggesting that these strains have more flexibility in trait correlations than the other strains in this study. Overall, the strongest departure from ancestral trait correlations was related to large shifts in correlation due to changes in a single trait (typically ROS).

Many of the specific changes to traits and trait relationships found in this study were strain- or population-specific. However, changes to ROS levels and correlations between other traits and ROS were common in FSH populations. FSC populations had lower ROS content than FSH populations (effect of FS temperature *F*_1,366_ = 10.70, *p* = 0.0012; mean relative ROS content for FSC = 0.071, mean for FSH = 0.133). The relationship between ROS content and growth temperature also differs between FSH and FSC populations on average, where the FSC populations have higher ROS content at 24°C than at 20°C, and FSH show the opposite pattern (interaction between growth temperature and FS temperature *F*_1,366_ = 4.545, *p* = 0.034). This is consistent with both warming below *T*_opt_ being sufficient to drive evolution, and with initial plastic increases in growth rates driving the eventual evolution of lowered growth rates due to increased pressure from faster metabolism [28–30].

The relationship between growth and outlier scores provides information about the potential fitness effects of evolving new trait relationships. We found that populations with large changes in trait correlations had up to 60% reduction in growth rates relative to their ancestral strain (electronic supplementary material, figure S10; *F*_1,55_ = 30.662, *p* < 0.01), even though these growth rates had stabilized by the end of the experiment. This indicates that while trait correlations evolve readily after population bottlenecks, large departures from ancestral trait correlations are likely to have a detrimental impact on growth rates. When FS populations were then grown for additional time (10 months with weekly transfers), growth rate did increase further, with a range of ±20% of ancestral growth. It is worth noting that the increase in growth rate occurred after long-term culturing under conditions that consistently favoured rapid population growth (batch culture) that far exceeded typical high growth periods for diatoms in nature. Typical diatom blooms may last weeks, and growth seasons for temperate diatoms typically span a few months. Taken together, our data show that departures from ancestral trait correlations, even if they are transient, can persist over periods of time spanning growth seasons. These deviations have the potential to play a role in determining evolutionary trajectories, as well as underlying variation in multi-trait phenotypes expressed early in adaptation.

## 3. Discussion

Observed variation in diatom phenotypes in the ocean is generated by a variety of evolutionary forces including natural selection and chance events. The relationships between the many traits that define these phenotypes can be assessed using a trait-scape. Here, six strains of the diatom genus *Thalassiosira* were diversified using repeated population bottlenecks and subsequently re-evolved as large populations. We demonstrate that novel phenotypes emerged, even in the absence of environmental change, and that multi-trait phenotypic evolution could be studied using reduced axes (a trait-scape). This suggests two fundamental insights. First, chance events such as repeated population bottlenecks can provide phenotypic variation, often associated with a drop in fitness, on which natural selection subsequently acts, whether or not the environment changes. This is likely to be important given the bloom and bust growth strategies of marine diatoms and the dynamic environments in which they live. The possible routes that environmentally driven evolution can take depend on which genetic and phenotypic starting points are available for selection to act on. We show that, in most cases, the bottlenecked populations simply reverted to their ancestral multi-trait phenotypes when subsequently evolved as large populations. However, different evolutionary starting points provided by bottlenecks do occasionally allow novel multi-trait phenotypes to evolve in large populations, even in a constant environment. A low proportion of bottlenecked populations evolved new multi-trait phenotypes, but when this happens repeatedly over many populations, as will be the case in the ocean, population bottlenecks could generate substantial diversity in diatom functional traits and their correlations. This is consistent with previous work which has shown that bottlenecks can generate diversity in other taxa where population size fluctuates, such as viruses and bacteria [31,32].

The second insight is that multi-trait variation is constrained to few fixed axes of variation. Specifically, we were able to define a trait-scape using a small number of orthogonal traits that captured all of the observed changes in the populations of multi-trait phenotypes throughout the experiment. This indicates that considering changes to multi-trait phenotypes made up of numerous intertwined functional traits can reveal patterns of phenotypic evolution, at least in the absence of dramatic or obviously stressful environmental change. This is useful because it does not depend on knowledge of how traits are linked to one or more other traits. This may be especially relevant for ecosystem modelling, where phytoplankton multi-trait phenotypes are often simplified using a master trait, which determines the values of all other functional traits via fixed trait correlations [5,6]. Here we demonstrate that, for our system, there is no master trait that can be used to understand how multi-trait phenotypes evolved—all of the traits projected strongly onto the two trait-scape axes.

In this study, repeatable multi-trait evolution occurred along a major axis of variation defined by several common diatom functional traits and their relationships. Because these functional traits are relevant for other phytoplankton functional groups [33], we expect the concept, utility and many of the relationships revealed in any given phytoplankton trait-scape to generalize to other phytoplankton, especially within functional groups (silicifiers, calcifiers). This is because all phytoplankton have similar functional traits, and many are linked to basic, conserved metabolism [34]. We do not expect that all functional groups have exactly the same trait-scape, as functional groups with different biogeochemical functions and environmental niches will most likely have different trait axes. However, trait-scapes can provide a reduced dimensional way to assess multi-trait phenotypic changes. Trait-scapes are also a useful because they provide a way to capture changes in trait correlations.

As mentioned above, ecosystem models often define phytoplankton phenotypes (or functional groups) using the relationships between the modelled traits and a master trait. Our findings suggest that master-trait approaches that rely on fixed correlations are inappropriate for studying multi-trait change within species or closely related species, and on this time scale in diatoms. Thus, this work calls for a reimagining of how we represent multi-trait phenotypes in models aimed at capturing phenotypic evolution. While additional work is needed to develop these new frameworks, our results suggest that tractable frameworks could be developed that leverage multivariate approaches to reduce the dimensions of variability (i.e. trait-scapes) while more accurately capturing how multi-trait phenotypes adapt.

Our study used two environments, both of which were nutrient replete and below the temperature optimum for these strains. While a similar degree of warming has been shown to drive adaptation in large laboratory populations of diatoms [17,35], major shifts in fundamental growth strategies, such as those associated with heat stress or nutrient starvation, would not be expected. While our FSH populations did not experience heat stress, an increase of 4°C is in line with the magnitude of change expected in many marine systems over the next 100 years [36]. This is also the magnitude of change that a phytoplankton population might experience due to mixing between adjacent water masses [37], or growing sequentially over several seasons [38]. Here we show that this degree of change is sufficient to select for novel phenotypes following diversification by bottlenecks.

While individual traits changed in response to warming, multi-trait phenotypes often returned to the ancestral location in the trait-scape. Some consistent patterns involving ROS content emerged in our study. High outlier scores for the multi-trait phenotype overall were often due to departures from ancestral correlations between ROS content and other traits. There is limited evidence that changes to cellular ROS content may be associated with evolutionary change, especially when population growth rate increases [28,30]. The consistency of ROS shifts was unusual; most other single trait changes were variable. For example, POC:PON evolved higher values in FSH populations than in FSC populations for strain 1587, while most genotypes show no difference (electronic supplementary material, figure S7). This suggests that for key traits, both the direction and the magnitude of evolutionary shifts differ between genotypes, especially when random processes contribute to evolution.

Based on the variation in changes to trait values across genotypes, and the prevalence of departures from ancestral trait correlations, we hypothesize that relative to individual trait values, multi-trait phenotypes and the variation in them defined by trait-scapes may be more stable across environmental gradients that do not provoke major shifts in growth strategies, at least at this level of taxonomic variation. Thus, trait-scapes can offer insight into fundamental constraints on functional trait variation within phytoplankton groups or closely related strains. However, the stability of the trait-scape in the face of novel or stressful environments is unknown, and should be explored in future studies.

## 4. Conclusion

Phytoplankton play crucial roles in aquatic food webs and biogeochemical cycling, and they do so through their interconnected web of functional traits. The trait-scape described by functional trait values and correlations in our study is robust to variation produced in the short term by chance events and natural selection, and across two similar and non-stressful environments. Most populations return to their ancestral locations in the trait-scape after demographic perturbations. However, a minority of populations evolve different multi-trait phenotypes from their own ancestors. Trait-scapes offer an alternative to master-trait-based views of how trait correlations constrain multi-trait phenotypes in diatoms, and we suggest that this same approach will also be useful in other phytoplankton functional groups. This multi-trait framework incorporates higher-level trait relationships and is a tractable way to understand and represent trait-based variation in phytoplankton.

## 5. Material and methods

### (a) Diatom cultures

Six strains of *Thalassiosira* sp. from the National Centre for Marine Algae and Microbiota at Bigelow were used: CCMP 1010, 1050, 1059, 1587, 2929 and 3367 (electronic supplementary material, table S1). These differed from each other in original isolation location, as well as traits such as cell size and population growth rate (see [9]. for a detailed description of the strains). Previous experiments using 13 different *Thalassiosira* strains including these six confirmed that the strains used in this study are a representative divergent sample of a larger collection of genotypes [9]. The culture collection is maintained at 20°C in the laboratory under conditions that are identical to the ancestral and FSC conditions during this experiment.

### (b) Culture maintenance

Cultures were grown in sterile f/2 media [39] made from natural seawater (collected in St Abbs, UK), at 20°C or 24°C and approximately 60 µmol photons m^−2^ s^−1^ (measured with a 4-pi sensor) at a 12 h : 12 h light : dark cycle. For the evolution experiment, cultures were maintained in transparent 48-well plates covered with Breathe-Easy breathable plate-seals (Sigma Aldrich). Ancestral populations were maintained under identical nutrient, light and temperature conditions, but in 40 ml culture flasks. Cultures were diluted every 7–10 days with fresh media with transfer sizes between 1000 and 2000 cells ml^−1^.

### (c) Evolution experiment

The experiment was divided into two phases, an initial three-month long reduced-selection (RS) phase (70–200 generations, at 20°C) followed by an eight-month full-selection (FS) phase (200–500 generations, at either 20°C or 24°C) (figure 1). The reduced-selection phase of the experiment was initiated with six replicates of each of six strains, resulting in 36 experimental populations. Three populations went extinct during the reduced-selection phase, so 33 populations entered the full-selection phase. The full selection was performed in a control (FSC, 20°C) and a warm environment (FSH, 24°C). Pilot studies determined that the warmed environment is below T_opt_ for these strains.

Trait measurements of populations were performed at the beginning of the experiment, at the end of the reduced-selection phase and at the end of the full-selection phase. During the experiment, trait values were measured under control conditions; at the end of the experiment, trait values were obtained under control (20°C) and high temperature (24°C) assay conditions. electronic supplementary material, table S2 lists which trait values were collected for each experimental stage. Analyses have a focus on the data collected under control conditions. Whenever data from high temperature assay conditions is analysed, this is stated.

#### (i) Reduced-selection phase

We repeatedly induced population bottlenecks (approx. eight cells transferred from the parent culture) in the 36 experimental populations. Initially, bottlenecks were induced every 7 days (approx. 13 generations). As growth rates decreased, this was increased to every 14 days. Populations were bottlenecked if they reached a minimum cell concentration of 2000 cells ml^−1^. Else, cultures were diluted to 500 cells ml^−1^, to allow for population recovery before a new bottleneck.

The bottlenecking was repeated five–nine times. This corresponds to a total experiment length of three months and 70–200 generations, depending on population growth rates. Towards the end of the bottlenecking phase, population growth had decreased to near zero. We define ‘near zero’ as populations showing no increase in fluorescence or visual evidence of viable cells over two weeks. In these cases the previous transfer was used to induce a new bottleneck. Growth rate was monitored via *in vivo* fluorescence. At the end of the RS phase (at the end of the series of bottlenecks), population growth rates were reduced by an average of 45% compared with ancestral growth rates (electronic supplementary material, figure S3).

#### (ii) Full-selection phase

Thirty-three populations entered this phase. During the full-selection phase, populations were propagated by batch culture with transfer sizes of approximately 1000 cells every 7 days, in either a control (20°C) or a warm environment (24°C). The warm environment caused a growth increase in ancestors, and did not kill the RS populations. In total, populations were transferred approximately 25 times in the full FS phase (200–500 generations). Population growth rates were measured every 5 to 10 transfers to monitor fitness recovery. Before the final trait measurements, the growth rate of all populations was measured over four transfers to ensure that the population growth rates had stabilized (electronic supplementary material, figures S4 and S5).

### (d) Trait measurements

Here, we measured a suite of traits that are typical in a phytoplankton ecophysiology study, and which are also amenable to being measured under conditions that allow a replicated evolution experiment to be carried out. See Argyle *et al.* [22] for a more detailed discussion of trait choice. To investigate how trait values changed throughout the experiment, trait measurements were performed at the onset of the experiment (A_0_), at the end of the reduced-selection phase (RS) and at the end of the full-selection phase (FSC, FSH). At the end of the full-selection phase, the experimental ancestors, which were maintained under control conditions throughout the experiment, were measured again to account for any trait change during normal culture maintenance (A_end_). Where applicable, all traits were measured during mid-exponential growth phase and daily mid-light phase. Single trait changes are in electronic supplementary material, figure S7. Trait measurements at the beginning and at the end of the reduced-selection phase included growth rate, cell size, cell complexity, chlorophyll a content, and particulate organic carbon and nitrogen content. Not all traits were measured at the end of the reduced-selection phase due to RS populations having very low growth rates, which limited the biomass available for measurements. At the end of the full-selection phase, a more extensive set of traits was measured for the FSC- and FSH-populations and the experimental ancestors, in both the control and the warm environment (electronic supplementary material, table S2). Previous studies with these populations showed that populations reach stable trait values after acclimatizing for 7 days to the test environment [9,22]. Therefore, trait measurements were performed for all populations after an acclimatization period of 7 days (one transfer) in the test environment, followed by transfer into new media, and measurement before reaching carrying capacity.

#### (i) Growth rate

Growth rates were measured via daily *in vivo* fluorescence using a plate reader (excitation: 455 nm, emission: 620 nm; Tecan Spark, UK). The exponential growth rate was calculated for each time step using

5.1 $$\mu =\frac{\ln(x_{2})-\ln(x_{1})}{t_{2}-t_{1}}.$$

Growth rates were determined based on the average growth over four consecutive time steps. During the bottleneck phase growth rates were determined based on single replicates per population; for the final trait measurements growth rates were determined using three replicates per population.

#### (ii) Cell size, cell complexity (granularity) and chlorophyll a

Cell size, cell complexity (granularity) [40] and chlorophyll a concentration were determined by flow cytometry. Cell size estimates were determined based on the median forward scatter and converted to cell diameter (μm) using a standard curve based on standard beads of 1, 3, 6, 10 and 25 µm beads as in [40]. Beads were used to calibrate cell size at each time point where it was measured, thus changes to the optical properties of cells will show up primarily as changes to the cell complexity measurement. Cell complexity (granularity) was estimated based on median side scatter, chlorophyll a (Chl a) was determined based on median fluorescence in the PerCP-Cy5-5-A channel (488 nm wavelength, filter 695/40BP).

#### (iii) Particulate organic carbon and nitrogen

Particulate organic carbon (POC) and nitrogen (PON) content were determined using elemental analysis. Populations were grown in 50 ml culture flasks containing 45 ml of media, up to a cell density of approximately 112 000 cells ml^−1^. The culture was split into two technical replicates and filtered onto pre-combusted Whatman GFF filters. Cell concentrations were determined using *in vivo* fluorescence and standard curves developed for each strain so that POC and PON content could be expressed per cell. Filters were dampened with 200 µl distilled water, and 100 µl 2 M hydrochloric acid was then applied to remove any inorganic carbon. After air-drying filters overnight, the samples were analysed in an elemental analyser (Thermo Fisher Scientific FlashSMART 2000 Elemental Analyzer).

#### (iv) Lipid content, silicic acid uptake and production of reactive oxygen species

The neutral lipid content, silicic acid uptake and production of reactive oxygen species were determined based on [22]. Neutral lipid content of cells was determined by adding a BODIPY 505/515 stain to the samples, at a final concentration of 2.6 µg ml^−1^. After 10 min incubation, samples were analysed in the flow cytometer using median FITC-A (488 nm wavelength, filter 530/30 BP).

Silicic acid uptake was calculated from PDMPO uptake by inoculating 500 µl samples with 0.125 µmol l^−1^ PDMPO dye (LysoSensor Yellow/Blue DND-160, Invitrogen) for 24 h; blank samples were inoculated in the same way excluding the dye. PDMPO is incorporated by cells with the same rate as silicic acid, allowing an estimate of the rate of silica production. After incubation under 12 h : 12 h light : dark conditions silicic acid uptake was determined using median AmCyan-A of the flow cytometer (405 nm wavelength, filter 525/50 BP), subtracting values of undyed samples from the dyed samples.

Relative intracellular ROS as a measure of stress was determined using H2DCFDA dye. The 500 µl sample volumes were inoculated for 5 h in the dark in the test environment (20/24°C) with a concentration of 80 µmol l^−1^ dye. Blank population samples excluding dye were inoculated in the same manner. After incubation, relative intracellular ROS was calculated from H2DCFDA fluorescence using a Tecan Spark plate reader (excitation: 488 nm, emission: 525 nm) and blank values were subtracted from the dyed sample values.

### (e) Data analysis

#### (i) Correction for influence of cell size on other traits

Cell size is known to influence many other functional traits; such as metabolic traits, which correlate with cell size [41]. This study included cells that spanned a size range of 5–25 µm in effective diameter; this means that for traits that scale with cell volume, variation in size can influence variation in those traits. Traits that were corrected for cell volume were granularity, Chl a, lipid content, ROS production, silicic acid uptake, POC and PON content, using equation (5.2). Growth rate was not corrected for cell volume, since the relationship between growth rate and cell size is not linear [41]. The equation to correct for the influence of cell size on trait values is given below.

5.2 $$\mathrm{Sample}\;\mathrm{trait}\;{\mathrm{value}}_{\mathrm{corrected}}=\frac{\mathrm{Sample}\;\mathrm{trait}\;{\mathrm{value}}_{\mathrm{original}}}{(4/3)\times \pi \times {\left(\mathrm{cell}\;\mathrm{diameter}[\mu m]/2\right)}^{3}}.$$

#### (ii) Principal component analysis

Principal component analyses (PCA) were performed after standardizing all trait data. The number of traits that were included in individual analyses differed, since not all traits and environments could be measured at all experimental stages (§5(d) and electronic supplementary material, table S2). This is because growth was very slow at the end of the RS phase of the experiment, which made obtaining sufficient biomass, or reasonably large signals relative to noise (both of which significantly increase uncertainty), not possible for all traits.

To assess movement of populations within the trait-scape, we generated the trait-scape first with the ancestral and FS trait data. Each trait (input variable) then becomes associated with a PCA score which can be used to project the RS populations onto the PCA axes. The trait-scape in this study does not contain redundant traits, with the exception of POC and PON. Specifically, the traits used for the PCA are largely orthogonal and so we do not believe that we have measured traits that are providing redundant information in this PCA. Thus, removing any traits from the PCA would alter the axes because the information provided by each trait is unique. One exception to this is that we keep both POC and PON in our analysis, as both are ecologically important traits.

#### (iii) Trait-scape connectivity

Ancestral, FSC and FSH locations were categorized as isolated, connected or novel (figure B1). To maximize the comparability between stages of the experiment, this categorization was performed on the PCA including trait values of ancestral and full-selection populations at 20°C. Trait values of RS populations were projected into the trait-scape. Below, ‘location’ always refers to the location of the populations in the trait-scape.

Populations that returned to the ancestral location in trait space were identified by calculating the Euclidean distance between ancestral RS locations, and the distance between ancestral and FS locations. If the latter distance was smaller than the former, the peak of the full-selection population was classified as an isolated fitness peak. For all isolated fitness peaks, a 75% confidence ellipse was calculated around the ancestral peak. All populations that did not meet the first criterion, but which fell within the 75% confidence ellipse of their ancestor were also classified as isolated fitness peaks. Any population that was not classified as an isolated fitness peak and that moved into the 75% confidence ellipse of another strain was classified as a connected fitness peak. All remaining unclassified populations were considered to have found a previously unoccupied location in trait-scape, classified as occupying a novel fitness peak.

#### (iv) Outlier analysis

To identify outliers among the FSC and FSH populations that diverged from ancestral trait values and trait correlations, all possible pairwise trait correlations were calculated for ancestors, FSC and FSH populations under control conditions. Ancestral trait correlations were calculated from linear regressions with 95% confidence intervals across all ancestral strains for each pairwise trait set (electronic supplementary material, figure S8). Residual values for FSC and FSH populations were calculated. Residual values of FSC and FSH populations that stayed within the ancestral confidence interval were set to zero for that trait correlation, because they did not significantly deviate from the pairwise trait correlation of the ancestral strains. Thus, only FSC and FSH residual values that fell significantly outside the ancestral correlation contribute non-zero values to the outlier scores. For each population an outlier score was calculated as the mean residual value across all pairwise trait correlations.

#### (v) Statistical analysis

To compare the intra- versus inter-specific variation in trait-scape, Euclidean distances between all ancestral, FSC and FSH populations were calculated based on their location in the trait-scape using Matlab. Using the resulting dataset, we calculated Euclidean distances within and between strains. Statistical analysis was then performed in the R environment using the nlme and lme4 packages [42]. We checked that residuals were normally distributed using a Q–Q plot; a generalized linear mixed-effects model was used to analyse data, with type of variation (inter- versus intra-specific) as fixed effect and strain as random effect. The effect of bottlenecking and backselection on growth rates was analysed using a generalized linear mixed-effects model. Experimental stage was included in the model as a fixed effect, and strain as a random effect. ANOVAs were used to estimate *p* values.

## Data Availability

All trait data can be found in the electronic supplementary material for this manuscript [43].
